# Adhesion Forces and Coaggregation between Vaginal Staphylococci and Lactobacilli

**DOI:** 10.1371/journal.pone.0036917

**Published:** 2012-05-18

**Authors:** Jessica A. Younes, Henny C. van der Mei, Edwin van den Heuvel, Henk J. Busscher, Gregor Reid

**Affiliations:** 1 Human Microbiology and Probiotics, Lawson Health Research Institute, London, Ontario, Canada; 2 Department of BioMedical Engineering, W. J. Kolff Institute, University Medical Center Groningen and University of Groningen, Groningen, The Netherlands; 3 Department of Epidemiology, University Medical Center Groningen and University of Groningen, Groningen, The Netherlands; 4 Departments of Microbiology & Immunology, and Surgery, University of Western Ontario, London, Ontario, Canada; Swiss Federal Institute of Technology Zurich, Switzerland

## Abstract

Urogenital infections are the most common ailments afflicting women. They are treated with dated antimicrobials whose efficacy is diminishing. The process of infection involves pathogen adhesion and displacement of indigenous *Lactobacillus crispatus* and *Lactobacillus jensenii*. An alternative therapeutic approach to antimicrobial therapy is to reestablish lactobacilli in this microbiome through probiotic administration. We hypothesized that lactobacilli displaying strong adhesion forces with pathogens would facilitate coaggregation between the two strains, ultimately explaining the elimination of pathogens seen *in vivo*. Using atomic force microscopy, we found that adhesion forces between lactobacilli and three virulent toxic shock syndrome toxin 1-producing *Staphylococcus aureus* strains, were significantly stronger (2.2–6.4 nN) than between staphylococcal pairs (2.2–3.4 nN), especially for the probiotic *Lactobacillus reuteri* RC-14 (4.0–6.4 nN) after 120 s of bond-strengthening. Moreover, stronger adhesion forces resulted in significantly larger coaggregates. Adhesion between the bacteria occurred instantly upon contact and matured within one to two minutes, demonstrating the potential for rapid anti-pathogen effects using a probiotic. Coaggregation is one of the recognized mechanisms through which lactobacilli can exert their probiotic effects to create a hostile micro-environment around a pathogen. With antimicrobial options fading, it therewith becomes increasingly important to identify lactobacilli that bind strongly with pathogens.

## Introduction

Vaginal and bladder infections are among the most common causes of illness in females. Antimicrobial treatment regimens have remained relatively static for 40 years without reducing recurrences, and now with drug resistance developing, efficacy is further diminishing. High throughput sequencing studies on well-characterized cohorts have revealed that *Lactobacillus iners, Lactobacillus crispatus, Lactobacillus gasseri*, and *Lactobacillus jensenii* dominate the vaginal microbiota in healthy women, but *L. crispatus* and *L. jensenii* are unable to withstand the influx of pathogens leading to infection, and thus are more displaced from the normal microflora [1]–[3]. This inability to persist is believed to be related to their lack of adhesion to the vaginal surface, other microbes and their failure to adapt to the changing urogenital environment [4]. Contrarily, the capacity of pathogens to adhere to each other and the mucosa is critical in the infection process [5], and of these organisms, aerobic *Escherichia coli* and the toxic shock syndrome (TSS) toxin-producing *Staphylococcus aureus* are the most virulent [6].

In patients with bacterial vaginosis, dense pathogenic biofilms cover the epithelial surface. Such biofilms afford not only a synergistic opportunity for survival and evasion of host defences, but also a means to resist host and exogenous antimicrobials, allowing the development of recalcitrant infections [7]. Interestingly, in a portion of women, bacterial vaginosis spontaneously resolves without antimicrobial intervention, and lactobacilli return to dominance [8]. *In vitro* studies have shown that the administration of certain probiotic lactobacilli can lead to disruption of these pathogenic biofilms [9], but the actual mechanism of interference and biofilm penetration has not been studied.

We hypothesized that probiotic lactobacilli used successfully to prevent recurrent infections, would display strong adhesion forces with pathogenic strains and be able to bind pathogens into coaggregates. If adhesion forces of lactobacilli with pathogens are greater than those binding the pathogens to each other, this could explain the disruptive process. The ability to penetrate dense pathogen biofilms could also be aided by biosurfactant production [10], but thereafter lactobacillus integration into the multilayered structure and formation of coaggregates with the pathogens would allow their antimicrobial molecules to disrupt the biofilms and reduce pathogen viability [9], [11], [12]. For instance, *Lactobacillus reuteri* RC-14 has shown the ability to penetrate mature *E. coli* biofilms and kill the *E. coli* upon coaggregation and integration with the biofilm [9].

Since the introduction of the atomic force microscope (AFM) and the development of techniques to prepare bacterial probes for interaction with surfaces [13]–[16], adhesion forces involved in bacterial (co)aggregation have been measured. Bacterial (co)aggregation has been demonstrated to be sensitive to even minor differences in adhesion forces between strains. Coaggregating and non-coaggregating oral bacterial pairs had adhesion forces of around 1 and 4 nN, respectively [17]. Aggregation between *Enterococcus faecalis* strains is mediated by the aggregation substance Agg, a plasmid encoded surface protein, and strains lacking Agg had smaller adhesion forces (1.3 nN) than strains possessing Agg demonstrating adhesion forces between 2.3 and 2.6 nN [18]. Moreover, adsorption of an antibody against Agg to an aggregating enterococcal strain reduced the adhesion force to around 1.2 nN. It has even been argued that a beneficial effect of cranberry juice on adhering urogenital pathogens could be attributed to a reduction in adhesion force between *E. coli* and a silicon nitride AFM tip upon adsorption of cranberry juice components from higher than 0.5 nN to smaller than 0.5 nN [19].

The aim of the current manuscript is to evaluate our hypothesis that adhesion forces mediating coaggregation between lactobacilli and staphylococci are stronger than the forces that mediate staphylococcal aggregation. Using AFM and coaggregation assays, we were able to demonstrate that lactobacilli indeed had equal or stronger adhesion forces to the staphylococci than the pathogens did with themselves, while furthermore pairs of strains showing more extensive (co)aggregation possessed significantly higher adhesion forces.

## Materials and Methods

### Bacterial Strains, Culture Conditions

Three TSS toxin 1–producing *S. aureus* strains, MN8 (isolated from a patient with menstrual TSS), COL (isolated from an operating theatre; a methicillin-resistant strain), and Newman (isolated from a human infection) were cultured aerobically from brain heart infusion (OXOID, Basingstoke, UK) agar plates in 10 ml of brain heart infusion broth at 37°C. Resident lactobacilli, *L. jensenii* RC-28, *L. crispatus* 33820, and the established probiotic strain *L. reuteri* RC-14 were cultured anaerobically from de Mann, Rogosa and Sharpe (MERCK, Darmstadt, Germany) agar plates in 10 ml de Mann, Rogosa and Sharpe broth at 37°C. All strains were harvested in late-exponential phase by centrifugation for 5 min at 5000 g at 10°C, washed twice with sterile phosphate-buffered saline (PBS: 150 mM NaCl, 10 mM potassium phosphate; pH 7.0) and resuspended in 2 ml of the same buffer.

#### Measurement of bacterial adhesion forces using atomic force microscopy

**Figure 1:**
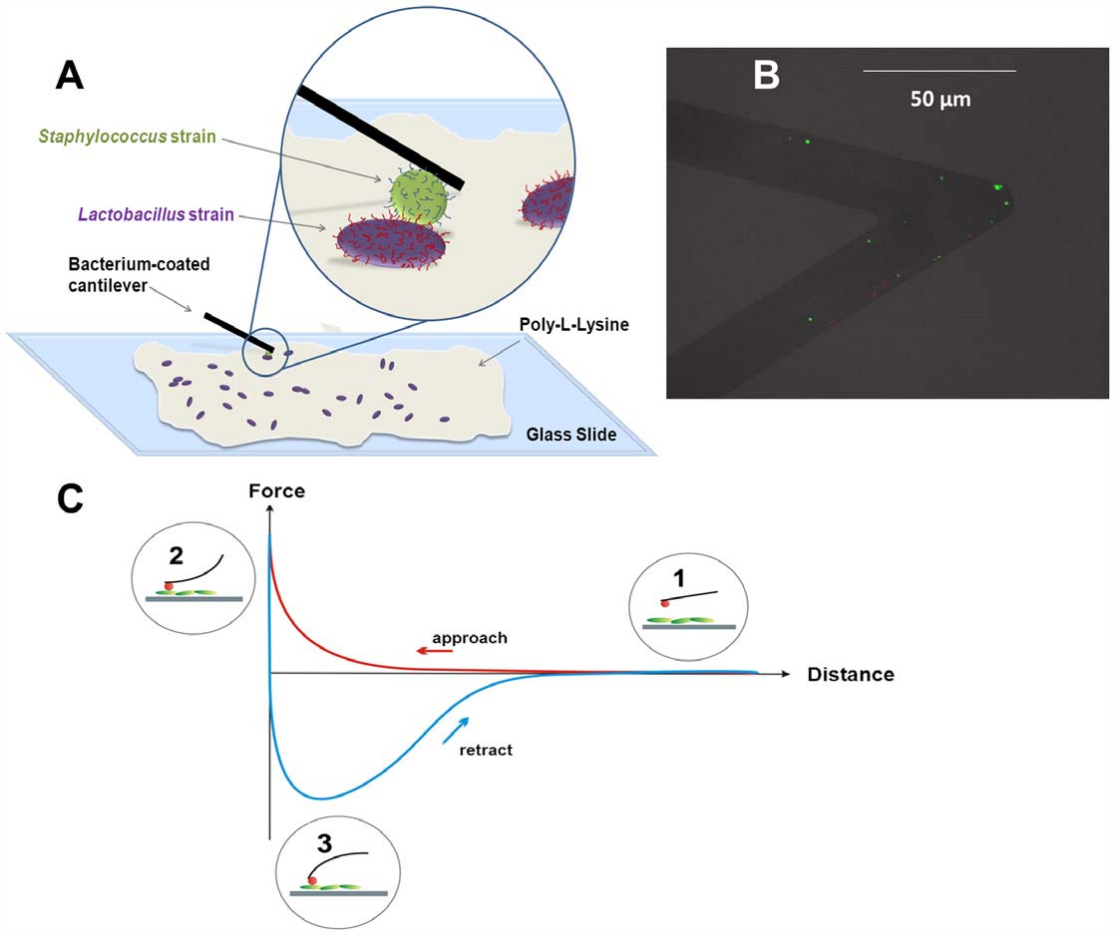
AFM experimental set-up and the resulting force-distance curve. **A**. Schematic presentation of the experimental AFM set-up depicting lactobacilli immobilized on poly-L-Lysine coated glass and a staphylococcus attached to the AFM cantilever. **B**. Fluorescence image of a bacterial probe coated with *S. aureus* Newman. The tipless cantilever (Bruker; Camarillo, CA) was prepared according to the methods outlined in the paper, used for AFM force adhesion experiments and stained using LIVE/DEAD *Bac*light viability stain (Molecular Probes Europe BV; Leiden, The Netherlands). Green spots represent viable bacteria. **C**. Schematic presentation of the cantilever deflection and the resulting force-distance curve upon approach and retraction of two bacteria in AFM. At large separation distances, no adhesion force is measured between bacteria (1), while at closer approach, repulsion occurs between the interacting bacteria, indicated by positive force values (2). Upon retraction, an attractive, negative force is measured (3).

In order to measure adhesion forces between bacterial pairs, a bacterium-coated AFM cantilever must be manoeuvred toward another bacterium that is immobilized on a substratum surface (Figure 1A and B), and the force upon approach and retraction of the bacterial probe is recorded from the cantilever deflection (see Figure 1C for a schematic example of a so-called “force-distance” curve). Upon approach, an increasing repulsive force is measured until physical contact, while upon subsequent retraction, an attractive force is found between the two adhering bacteria until failure [20]–[22].

#### Immobilization of bacteria

Glass slides were used for the immobilization of bacteria. The glass surface was cleaned by sonication for 2 min in 2% RBS35 and subsequently thoroughly rinsed with demineralized water, 70% ethanol, and finally with demineralized water. After air-drying, 20 µl of a 0.1% w/v poly-L-lysine solution (Sigma-Aldrich, Zwijndrecht, The Netherlands) was put on the glass surface and allowed to air dry. Bacterial suspensions of 10^5^ bacteria/ml (lactobacilli) and 10^6^ bacteria/ml (staphylococci) were vortexed with a bench-top vortex for 5 s before a 50 µl suspension droplet was added to the glass slide, yielding a low number of bacteria of between 10^3^ and 10^4^ bacteria/cm^2^, respectively. A low number of adhering bacteria was preferred in order to enable the selection of single immobilized bacteria for force measurements. After 20 min, the slide surface was carefully rinsed with demineralized water to remove any loosely adhering bacteria and transported in a covered petri dish containing paper moistened with demineralized water. All surfaces with immobilized bacteria were freshly prepared for each experiment.

#### Preparation of AFM probes

Bacterial AFM probes were prepared by adhering bacteria to “V”-shaped tipless AFM cantilevers (Veeco, DNP-0, Woodbury, NY, USA) through the use of a micromanipulator (Narishige International, Tokyo, Japan) under microscopic observation (Leica DMIL, Wetzlar, Germany). A small droplet of poly-L-lysine solution was placed on a glass slide, and the cantilever was dipped in the droplet for 2 min. After a 2 min drying period, the cantilever was dipped in a bacterial suspension (10^5^ bacteria/ml for lactobacilli and 10^6^ bacteria/ml for staphylococci) for 2 min and placed in a covered box with wet paper on the side to ensure moist conditions during transport to the AFM (note that all bacterial probes were freshly prepared for each experiment). Bacterial strains were grouped into identical pairs of staphylococci (S-S), and mixed pairs of lactobacilli and staphylococci (L-S). Staphylococci were the preferential probe bacteria, because of their spherical shape.

#### Bacterial adhesion force measurements

AFM measurements were carried out at room temperature in sterile PBS (pH 6.0) using an optical lever microscope (Nanoscope V, Digital Instruments, Woodbury, NY, USA) with *z*-scan rates of 1.0 Hz under a maximal loading force of 5 nN. Calibration of cantilevers was performed using the AFM Tune-it Version 2 software, yielding an overall average spring constant of 0.053±0.003 Nm^−1^. Force curves were measured after different contact times (0, 30, 60, and 120 s) between the bacterial probe and a bacterium immobilized on the glass slide and the maximal adhesion force upon retraction was recorded. All force curves were analyzed using Force-Distance software (Version 3.0.0.19).

In order to verify that a bacterial probe enabled a single contact with the surface, a scanned image in AFM contact mode with a loading force of 1–2 nN was made at the onset of each experiment and examined for double contour lines, which are indicative of multiple bacteria on the probe in contact with the bacterium selected on the slide. Any probe exhibiting double contour lines were discarded. At this point it must be noted however, that double contour line images seldom or never occurred, since it represents the unlikely situation that bacteria on the cantilever are equidistant to an immobilized bacterium on the glass surface within the small range of the interaction forces, which is unlikely if only by the angle under which the cantilever is in contact with the substratum. To ensure that a bacterial probe was not affected by previous measurements, a force curve at 0 s surface delay on clean glass was compared to five initially measured control force curves on glass. If the continued measurement differed by more than 0.2 nN from the average control force, data were discarded and a new probe prepared. For each combination of bacterial strains, at least 40 force-distance curves were recorded with two to four bacterial probes and bacteria from at least two different cultures of each strain.

### Coaggregation Assay

Bacterial suspensions in PBS (pH 6.0) of all strains were adjusted to equal concentrations of bacteria, after which equal volumes of each pair were mixed for 20 s using a bench top vortex, and left for 2 h. A droplet of this suspension was then put on a glass slide and Gram-stained for visual observation of aggregates, defined as visible clumps of bacteria and classified according to the presence of large and dense visible clumps of bacteria (++), small and sparsely distributed clumps (+), or no visible clumps or bound bacteria (−), as based on the assays used by Kolenbrander *et al*. for oral-co-aggregation [23].

### Statistical Analysis

In order to determine the statistical significance of differences in maximal adhesion forces between L-S and S-S pairs, the differences in adhesion force between S-S pairs and the corresponding L-S pairs were calculated for each pair of *Lactobacillus* and staphylococcal strains at all surface delay times up to 120 s using a linear mixed model (LMM). For a fixed combination of strains, *i.e.* a specific L-S or S-S pair, the LMM was applied to account for random variations due to the differences in adhesion forces, including the use of multiple cultures and probes. Next, procedure MIXED of SAS (Version 9.2) using restricted maximum likelihood and the Kenward-Rogers option for the number of degrees of freedom was used to fit the LMM. This yielded modeled values of the mean adhesion forces at all four surface delay times, representing the maximal adhesion force from the interaction of each bacterial pair as can be read from Figure 1C at position 3.

The null-hypothesis that the mean adhesion forces for the L-S pairs equals the corresponding S-S pairs at all four surface delay times was tested for each mixed pair separately with an F-test at significance level *α*  = 0.05.

The mean adhesion forces from this LMM at 120 s were used as input for the two-sample t-test to investigate a relation between adhesion forces and coaggregation scores and statistical significance was set at p<0.05.

## Results

**Figure 2:**
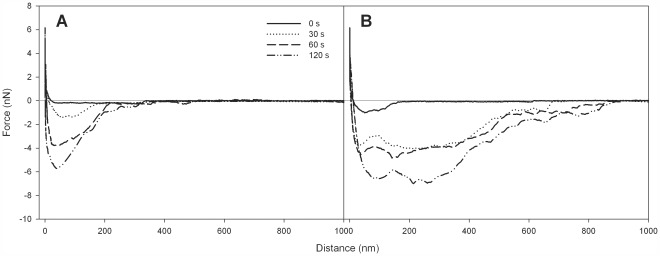
AFM force-distance curves from different bacterial pairs. **A**. Representative force-distance curves between an identical pair of *S. aureus* Newman with retraction curves measured after 0, 30, 60, and 120 s surface delay. **B**. Representative force-distance curves between a mixed pair of *L. crispatus* 33820 and *S. aureus* Newman. Retraction curves were measured after 0, 30, 60, and 120 s surface delay time.

Figure 1A and C show schematics of the experimental AFM setup used in this study and the force-distance data that is generated. Examples of actually measured force distance curves over the surface delay time points are shown in Figures 2A and B for an identical staphylococcal pair and a mixed pair of staphylococci and lactobacilli, respectively. For both bacterial pairs, adhesion forces increased over time. For the identical staphylococcal pair (Figure 2A), a maximal adhesion force of −5.8 nN was reached after 120 s, which is considerably smaller than the maximal adhesion force of −7.1 nN between the mixed *Staphylococcus* and the *Lactobacillus* strain (Figure 2B). The maximal adhesion force was reached at slightly closer distances for the identical staphylococcal pair (40 nm) compared to its mixed counterpart (70 nm), but pull-off events were sustained at much larger distance ranges for the mixed pair (>200 nm).

**Figure 3:**
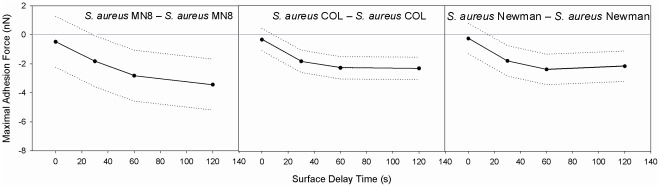
Maximal adhesion forces as a function of surface delay. Maximal adhesion forces, obtained using the LMM on the measured data, as a function of the surface delay time for the three strains of identical staphylococcal pairs involved in this study, with their 95% confidence intervals indicated by the dotted lines.

Figure 3 summarizes the maximal adhesion forces derived from the LMM as a function of the surface delay times for the three identical S-S pairs in this study. *S. aureus* MN8 consistently showed the strongest adhesion forces irrespective of the surface delay time, increasing from −0.5 to −3.4 nN within 120 s. *S. aureus* COL and Newman displayed similar increases in adhesion forces over time, but adhesion forces were generally smaller than those of MN8. Note, from Figures 2A and B, that depending on the bacterial combination involved, an increase in surface delay time may be accompanied by adhesion forces sustaining over longer distances.

**Figure 4:**
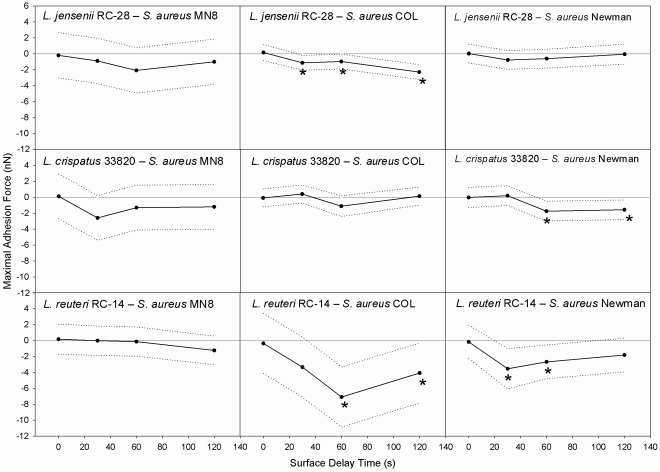
Maximal adhesion force differences between L-S and S-S pairs as a function of surface delay. Differences between the maximal adhesion forces for mixed pairs of staphylococci and lactobacilli pairs (L-S) and the corresponding identical staphylococcal pairs (S-S) as a function of the surface delay time, together with their 95% confidence intervals indicated by the dotted lines. Positive values indicate stronger adhesion forces between identical S-S pairs than between mixed L-S pairs. Significant differences (confidence interval not including the zero line) from the corresponding S-S pair at individual time points are indicated by an asterisk (*).

The difference in maximal adhesion forces between mixed pairs of staphylococci and lactobacilli and each corresponding S-S pair is shown in Figure 4 as a function of surface delay time. The LMM analysis for all time points indicated that the L-S pairs had equal or stronger adhesion forces when compared to the S-S pairs. While the L-S pairs on the whole showed stronger adhesion forces (2.2–6.4 nN) than the S-S pairs (2.2–3.4 nN) after 120 s, there were differences seen within the lactobacilli strains themselves. Pairs involving the probiotic strain *L. reuteri* RC-14 after 120 s surface delay, overall had the strongest adhesion forces (4.0–6.4 nN) and showed significant differences in adhesion forces with two pathogen pairs (p<0.05). On the other hand, *L. jensenii* RC-28 and *L. crispatus* 33280, both vaginal residents showed statistically significant different adhesion forces with only one pathogen pair each (p<0.05). One noteworthy point is that there were no significant differences between L-S pairs with the *S. aureus* MN8 strain; all such pairs contained either *S. aureus* COL or Newman.

**Figure 5:**
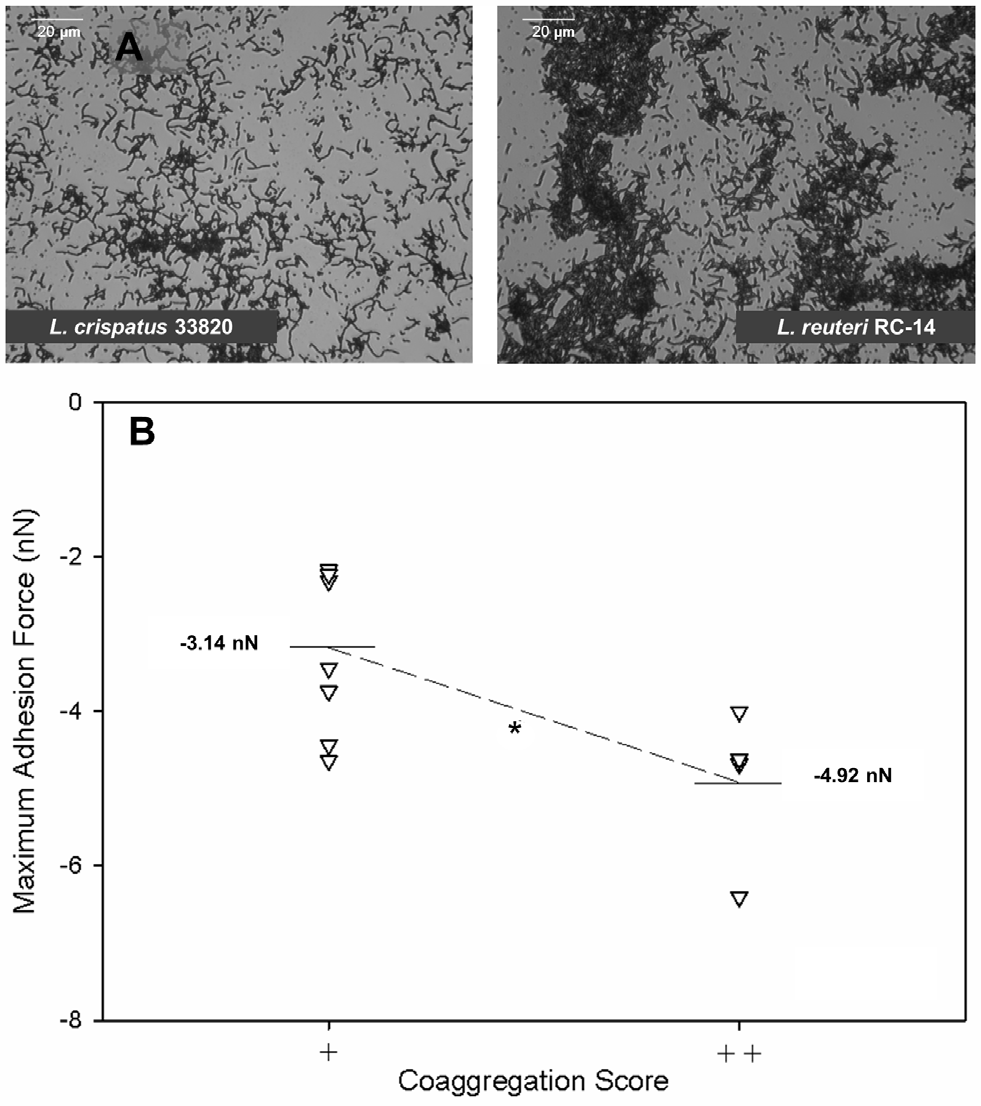
The relationship between coaggregation scores and maximum adhesion forces. **A**. Phase-contrast micrographs, demonstrating the difference between coaggregation score (+) and (++) for mixed pairs of *L. crispatus* 33820 (left) and *L. reuteri* RC-14 (right) with *S. aureus* COL. Images were obtained with a CCD camera (Basler AG, Germany) mounted on a phase contrast microscope (Leica DM2000; Leica Microsystems Ltd, Germany) set at 40× objective. **B**. Maximal adhesion forces grouped according to the corresponding coaggregation scores for the identical and mixed bacterial pair included in this study. The adhesion forces shown here are the means of the two groups of coaggregation scores, also visually represented with the thin grey horizontal lines. The dotted grey line visually represents the difference between the mean of the maximal adhesion forces of the two coaggregation groups, at a statistically significant difference of p  = 0.020 (*).

Within all pairs studied, only coaggregation scores ++ (dense clumping for *L. reuteri* RC-14 with *S. aureus* COL) or + (minimal clumping as seen for *L. crispatus* 33820 with *S. aureus* COL) were observed (Figure 5A). The pairs were grouped according to their coaggregation scores and their corresponding maximal adhesion forces (Figure 5B). Pairs that coaggregated well (++) had a significantly (p  = 0.020) higher adhesion force (−4.9±1.0 nN) than pairs coaggregating less well (+), which had an adhesion force of −3.1±1.1 nN.

## Discussion

Bacterial adhesion is an important determinant of biofilm formation on host surfaces and their pathogenesis. Biofilms can be formed by adhesion of single bacteria or coaggregates, emphasizing the role of adhesion forces between bacteria in the infectious process. In this study, we hypothesized that the adhesion forces between lactobacilli and pathogenic staphylococci would be stronger than between staphylococcal pairs. Furthermore, we proposed that these adhesion forces may mediate coaggregation. Indeed the main findings of this study supported our hypotheses. Adhesion forces between pairs of lactobacilli and staphylococci were equal or greater in magnitude than adhesion forces between staphylococcal pairs in all cases (Figure 4). In addition, a significant difference was found between the adhesion forces corresponding to the two coaggregating scores (p = 0.020). Statistical significance is generally hard to establish in the measurement of adhesion forces between microorganisms using AFM. Both parametric and non-parametric statistics as well as Weibull analyses of adhesion forces have been applied to compare AFM adhesion forces in biological systems [24]. The LMM statistical method [25], [26] applied here is relatively new in the field and allows the user to combine and account for multiple sources of variation (inter-probe and intra-probe effects for specific pairs) in order to be able to identify the true standard error around the mean adhesion forces between pairs at different time points. Therewith LMM has allowed us to determine realistic statistical significances, instead of using statistical tests that cannot incorporate this heterogeneity and thus provide either over- or under-estimates of standard errors. This type of statistical model is highly recommended for adhesion force data, as it is able to deal with issues that have hitherto compounded the analysis of this type of heterogeneous data.

Adhesion forces between lactobacilli and pathogenic staphylococci occurred instantly upon contact and matured within one to two minutes. Clinically, it has been shown that the microbiota can shift within days from being healthy and dominated by two of the species tested here, *L. crispatus* or *L. jensenii* [1]–[3], to a pathogen-dominated aberrant microbiota, including in some women to one that has a large abundance of *S. aureus* (unpublished data). The adhesion forces of the *S. aureus* strains with *L. crispatus* 33820 and *L. jensenii* RC-28 were less strong when compared to those with the probiotic RC-14. This suggests that *Lactobacillus* species effective in displacing pathogens, need to display strong adhesion forces to their pathogen targets, a desirable probiotic characteristic for infectious interventions. As studies of the human microbiome divulge, critical changes that lead to homeostasis and health or disease, the ability to understand physical forces at the nano-level that influence these dynamic reactions, will be critical to develop novel therapeutic interventions.

Although all three *S. aureus* strains involved in this study have the ability to produce toxic shock syndrome toxin 1, only *S. aureus* MN8 was isolated from a clinical case of TSS. Interestingly, *S. aureus* MN8 had stronger adhesion forces to itself when compared to *S. aureus* Newman and COL (see Figure 3), especially after 120 s surface delay that could hardly be surpassed by lactobacillus interaction with *S. aureus* MN8 (see Figure 4). This suggests that strong adhesion forces between pathogenic organisms must be considered as a virulence factor.

Coaggregation between enterococci and different oral bacterial pairs has been previously shown to occur with forces ranging from 2.6 to 4.0 nN [17], [18], which are slightly less than observed here, probably because we account for bond-strengthening while most other studies only report adhesion forces immediately upon contact. Adhesion forces between bacteria can become stronger over time due to progressive removal of water from in between the interacting cell surfaces, re-arrangement of surface structures and unfolding of binding molecules. Note that these aspects of bond-strengthening are all physico-chemical in nature and occur for inert polystyrene particles as well. Irreversible anchoring of organisms, through, for example, production of extracellular polymeric substances, requires more time and is unlikely to happen in the absence of nutrients, as applied in the current study which occurred in PBS. Interestingly, bond-strengthening was accompanied by adhesion forces extending over longer distances, which is likely due to the involvement of more and longer adhesive cell surface appendages in the bond after strengthening. This opens an alternative way to analyze our data.

The analysis presented so far is based on adhesion forces, whereas the area under a force-distance curve represents the energy required to disrupt the bond between two bacteria. Analysis of the data in terms of adhesion energy (see File S1 and Figure S1) demonstrated that overall the L-S pairs had greater or equal adhesion energies than the S-S pairs and four L-S pairs showed significantly stronger adhesion energies (p<0.05) than their corresponding S-S pairs, in general agreement with our hypothesis and the analysis on basis of adhesion forces. However, whereas adhesion forces confirmed our hypothesis for four L-S pairs (Figure 4), the same number of L-S pairs also showed significantly greater adhesion energy than the S-S pairs. Only one pair was different in bacterial strains from the adhesion force analysis: the analysis on the basis of adhesion energies identified significant effects for *L. jensenii* RC-28 and *S. aureus* MN8, while in the adhesion force analysis, *L. jensenii* RC-28 was significant with the pathogen *S. aureus* strain COL. The combined analysis of adhesion forces and energies strengthens our conclusions, despite the strain difference detected between both analyses.

The coaggregation process in the oral cavity has been well described not only for diseased states but also for the maintenance of a relatively homeostatic microbiota [27]. In the vagina, when the coaggregates are pathogen-dominated, conditions like bacterial vaginosis arise and increase the subject’s risk of numerous complications including infections and preterm labour [28]. On the other hand, when lactobacilli form coaggregates and bind to pathogens, this results in a return to homeostasis [29], as coaggregation creates a hostile biochemical micro-environment around a pathogen and prevents it from continuation of growth and domination of the niche. Therewith this study provides support for the use of probiotic lactobacilli to treat and prevent the most common aberrant conditions in women, namely urogenital infections.

In summary, adhesion forces between lactobacilli and three virulent toxic shock syndrome toxin 1–producing *S. aureus* strains, were found to be equal or stronger than between staphylococcal pairs, especially for the probiotic *L. reuteri*. In addition, pairs of strains showing stronger adhesion forces showed more extensive (co)aggregation. The lower adhesion forces between resident lactobacilli and pathogenic staphylococci may explain why these lactobacilli are more easily displaced by urogenital pathogens *in vivo* [28]. Therewith, this study opens a new pathway for the design of effective probiotic strains that involves optimization of the adhesion forces governing coaggregation with target pathogens. AFM, as applied here, can provide a quantitative means to guide this process.

## Supporting Information

Figure S1 **Mean adhesion energy differences between L-S and S-S pairs as a function of surface delay.** The differences for the mixed pairs of staphylococci and lactobacilli pairs (L-S) and the corresponding identical staphylococcal pairs (S-S) are shown here with their 95% confidence intervals (dotted lines). Positive values indicate higher adhesion energy for an identical S-S pairs than for the mixed L-S pair. Significant differences (confidence interval not including the zero line) from the corresponding S-S pair at individual time points are indicated by an asterisk (*).
(TIF)

File S1 **Analysis based on adhesion energy.**
(DOC)
