# Supplementary figures and images for: Adhesion Forces and Coaggregation between Vaginal Staphylococci and Lactobacilli
Source: PLoS One. 2012 May 18;7(5):e36917. doi: 10.1371/journal.pone.0036917 (PMC3356358; doi:10.1371/journal.pone.0036917)

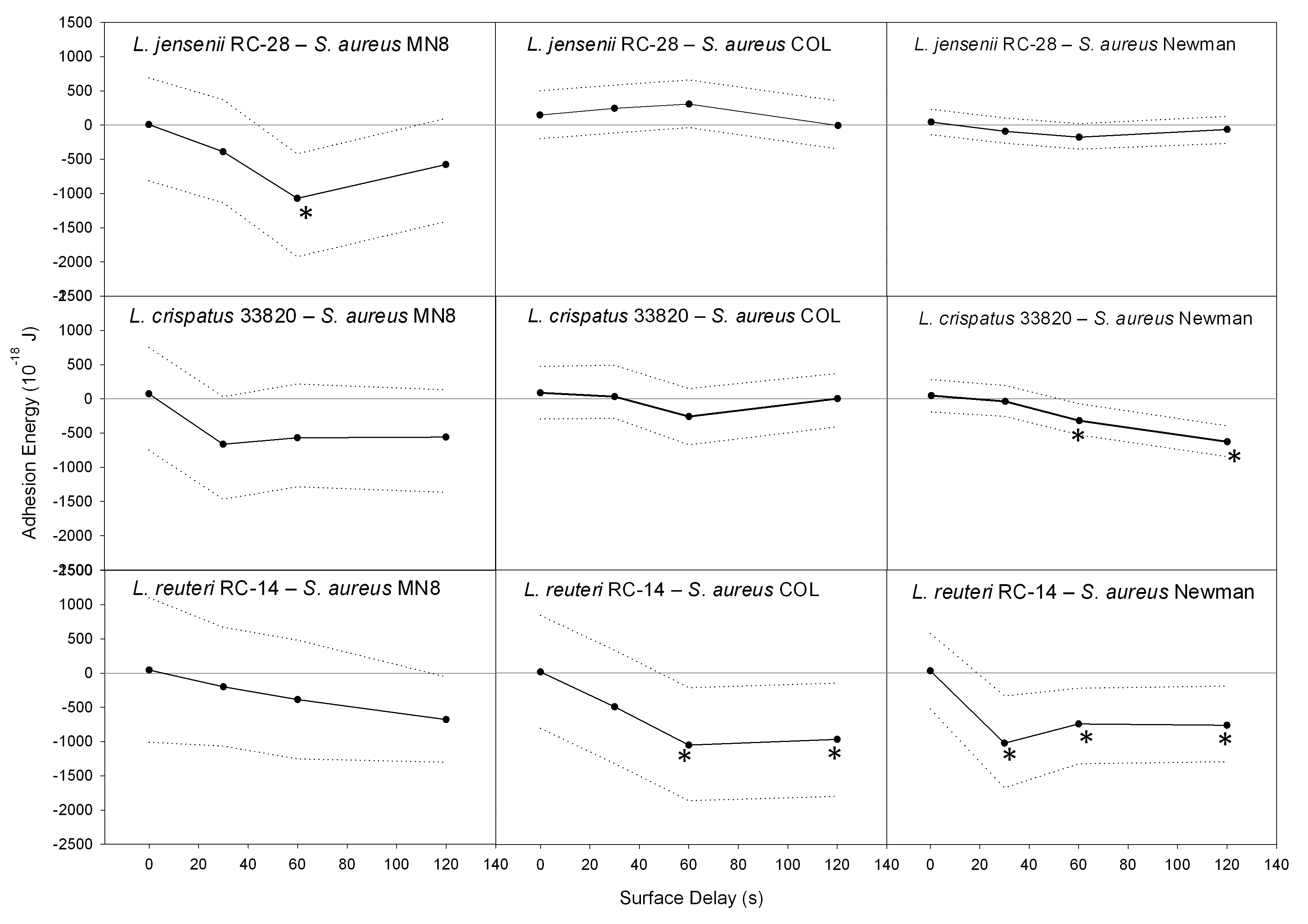

Supplement: Figure S1 — Mean adhesion energy differences between L-S and S-S pairs as a function of surface delay. The differences for the mixed pairs of staphylococci and lactobacilli pairs (L-S) and the corresponding identical staphylococcal pairs (S-S) are shown here with their 95% confidence intervals (dotted lines). Positive values indicate higher adhesion energy for an identical S-S pairs than for the mixed L-S pair. Significant differences (confidence interval not including the zero line) from the corresponding S-S pair at individual time points are indicated by an asterisk (*). (TIF) [file pone.0036917.s001.tif]
