# Supplementary material for: Adhesion Forces and Coaggregation between Vaginal Staphylococci and Lactobacilli
Source: PLoS One. 2012 May 18;7(5):e36917. doi: 10.1371/journal.pone.0036917 (PMC3356358; doi:10.1371/journal.pone.0036917)
Supplement: File S1 — Analysis based on adhesion energy. (DOC) [file pone.0036917.s002.doc]

**Supplementary Material S1: Analysis based on adhesion energy**

From each force-distance curve, the adhesion energy was determined from the area under the retract force-distance curve and statistically analyzed exactly as described for the adhesion forces.

At all-time points, L-S pairs had similar or greater adhesion energies (-39 to -1689 x 10^-18^ J) than corresponding identical S-S pairs (-96 to -762 x 10^-18^ J) with the exception of the pair *Lactobacillus* *jensenii* RC-28 with *Staphylococcus aureus* COL. Figure S1 shows the difference in adhesion energy between the L-S and their corresponding S-S pairs as a function of surface delay time. Analysis demonstrated significantly (p < 0.05) higher adhesion energies for four L-S pairs at various surface delays as compared to corresponding S-S pairs, with pairs involving *L*. *reuteri* RC-14 possessing the highest adhesion energies after bond-strengthening (-1015 to -1565 x 10^-18^ J).

The L-S pairs demonstrating significantly higher adhesion energies with respect to corresponding S-S pairs were identical to the pairs showing significantly stronger adhesion forces with the exception of the pair *L. jensenii* RC-28 with *S. aureus* MN8. For this pair, significant (p < 0.05) differences were demonstrated in the energy analysis, while pairs of *L. jensenii* RC-28 showed significantly stronger adhesion forces than when paired with *S*. *aureus* Newman.

Overall, the adhesion energy analysis strengthens the conclusion based on a force analysis that L-S pairs of both probiotic and resident lactobacilli adhere and bind to pathogens more tenaciously than the pathogens do to themselves.

**Figure S1.** **Mean adhesion energy differences between L-S and S-S pairs as a function of surface delay.** The differences for the mixed pairs of staphylococci and lactobacilli pairs (L-S) and the corresponding identical staphylococcal pairs (S-S) are shown here with their 95% confidence intervals (dotted lines). Positive values indicate higher adhesion energy for an identical S-S pairs than for the mixed L-S pair. Significant differences (confidence interval not including the zero line) from the corresponding S-S pair at individual time points are indicated by an asterisk (*).
